# Making Home Safe for Children

**Published:** 2012-03-01

**Authors:** Jamshed Akhtar

**Affiliations:** Department of Pediatric Surgery, National Institute of Child Health Karachi, Pakistan

Trauma is an important cause of mortality and morbidity in children. A significant number of injuries occur in home environment [1]. This subject has been studied extensively in western countries and huge data is generated. Based upon the findings many policy decisions have been taken in order to minimize the potential threats at home. In addition many educational activities are conducted for parents to teach safe practices for preventing accidents at home [2]. There are few reports available on the subject from Pakistan, though most not conducted on population basis [3,4]. Many of these are based upon hospital emergency room records that may not reflect a true picture of the magnitude.

It is important to know the pattern of trauma in children from developing countries as significant differences exist in socio-economic pattern and government regulatory policies in comparison with Untied States and European union [5,6]. In this issue of APSP Journal of Case Reports, Mirza et al reported a 6 month old baby who ingested crystal gel ball, being given by other child. The patient died in post operative period [7]. It is important to report this case to higher authorities as this involves putting a ban on import of such hazardous products from other countries. It would be of interest to note that a ban is already in place on this product in Italy and probably other European countries, as its potential threats have been reported by consumers’ societies [8].

Physicians involved in care of children thus have moral responsibility to address this issue on priority basis. In this regard steps must be taken in educating public through seminars and talk shows. Involving members of civil society groups and use of electronic media, which is quite influential in this regard, shall boost the momentum. Health officials must be taken on board for implementation of policy decisions through legislation. Preventing children from accidents should therefore be a priority in health related policies.

## Footnotes

**Source of Support:** Nil

**Conflict of Interest:** None declared

## References

[1] ( 2010). Litovitz T, Whitaker N, Clark L. Preventing battery ingestions: an analysis of 8648 cases. Pediatrics.

[2] Reich S M, Penner E K, Duncan G J (2011). Using baby books to increase new mothers' safety practices. Acta Pediatr.

[3] Zia N, Khan U R, Razzak J A, Puvanachandra P, Hyder A A (2012). Understanding unintentional childhood home injuries: pilot surveillance data from Karachi, Pakistan.. BMC Research Notes.

[4] ( 2004). Razzak JA, Luby SP, Laflamme L, Chotani H. Injuries among children in Karachi, Pakistan--what, where and how. Public Health.

[5] Hyder A A, Sugerman D E, Puvanachandra P, Razzak J, El-Sayed H, Isaza A (2009). Global childhood unintentional injury surveillance in four cities in developing countries: a pilot study. Bull World Health Organ.

[6] Fatmi Z, Kazi A, Hadden W C, Bhutta Z A, Razzak J A, PappaG (2009). Incidence and pattern of unintentional injuries and resulting disability among children under 5 years of age: results of the National Health Survey of Pakistan.. Paediatr Perinat Epidemiol.

[7] Mirza B, Sheikh A (2012). Mortality in a case of crystal gel ball ingestion: An alert for parents. APSP J Case Rep.

[8] Product recalls.

